# MathDAMP: a package for differential analysis of metabolite profiles

**DOI:** 10.1186/1471-2105-7-530

**Published:** 2006-12-13

**Authors:** Richard Baran, Hayataro Kochi, Natsumi Saito, Makoto Suematsu, Tomoyoshi Soga, Takaaki Nishioka, Martin Robert, Masaru Tomita

**Affiliations:** 1 Institute for Advanced Biosciences, Keio University, Tsuruoka, Yamagata 997-0017, Japan; 2 Department of Biochemistry and Integrative Medical Biology, School of Medicine, Keio University, Shinanomachi, Shinjuku-ku, Tokyo 160-8582, Japan; 3 Present address: Institute of Chemistry, Slovak Academy of Sciences, Dúbravská cesta 9, 845 38 Bratislava, Slovakia

## Abstract

**Background:**

With the advent of metabolomics as a powerful tool for both functional and biomarker discovery, the identification of specific differences between complex metabolite profiles is becoming a major challenge in the data analysis pipeline. The task remains difficult, given the datasets' size, complexity, and common shifts in migration (elution/retention) times between samples analyzed by hyphenated mass spectrometry methods.

**Results:**

We present a Mathematica (Wolfram Research, Inc.) package MathDAMP (Mathematica package for Differential Analysis of Metabolite Profiles), which highlights differences between raw datasets acquired by hyphenated mass spectrometry methods by applying arithmetic operations to all corresponding signal intensities on a datapoint-by-datapoint basis. Peak identification and integration is thus bypassed and the results are displayed graphically.

To facilitate direct comparisons, the raw datasets are automatically preprocessed and normalized in terms of both migration times and signal intensities. A combination of dynamic programming and global optimization is used for the alignment of the datasets along the migration time dimension.

The processed datasets and the results of direct comparisons between them are visualized using density plots (axes represent migration time and m/z values while peaks appear as color-coded spots) providing an intuitive overall view. Various forms of comparisons and statistical tests can be applied to highlight subtle differences. Overlaid electropherograms (chromatograms) corresponding to the vicinities of the candidate differences from any result may be generated in a descending order of significance for visual confirmation. Additionally, a standard library table (a list of m/z values and migration times for known compounds) may be aligned and overlaid on the plots to allow easier identification of metabolites.

**Conclusion:**

Our tool facilitates the visualization and identification of differences between complex metabolite profiles according to various criteria in an automated fashion and is useful for data-driven discovery of biomarkers and functional genomics.

## Background

The identification of specific differences between metabolite profiles plays a prominent role in metabolomic data analysis and can be useful for the discovery of biomarkers or the characterization of specific biological activities. Hyphenated mass spectrometry methods (GC-MS, LC-MS, CE-MS, etc.) are among the most common analytical tools for metabolomics. Most produce large datasets that are not easily interpretable using the software provided by most instrument manufacturers. The common data analysis workflow, starting from raw data, usually includes the detection of peaks, their integration, matching of corresponding peaks across datasets and subsequent multivariate analysis [1]. Several tools enabling automation of the procedure are available [2-7], but the overall task still proves challenging given the datasets' size, complexity, common shifts in migration times between datasets, and the need to identify metabolites. In addition, some of these tools either provide only partial solutions (generation of integrated peak lists) or were developed for a specific type of analysis (e.g. GC-MS) and some alignment algorithms may not be very robust when migration time differences are large and the composition of samples is highly variable. Moreover, automated peak picking and integration remains an important challenge that is complicated by the wide range of peak intensities, sometimes poor separation of compounds and the resulting distorted peak shapes, leading to multiple incorrect assignments of differences. While visual exploration of the raw data has been used to complement automated data analysis [8], this often comes at the expense of convenience and versatility. Direct chromatogram comparisons bypass peak picking and integration to select areas of interest from raw data or to locate differences between metabolite profiles [9]. To apply direct chromatogram comparisons as a complement or an alternative to the multivariate analysis of integrated peak lists, automation of the processing of raw data along with suitable visualization and metabolite identification methods are desirable. With MathDAMP, we provide a complete series of such tools, capable of providing an overall view of the differences between metabolite profiles according to different criteria. The functionality of the package is demonstrated with CE-MS data, which is particularly challenging due to the more significant migration time shifts, but the tools can be used for other types of hyphenated mass spectrometry methods as well.

## Implementation

Differences between metabolite profiles in MathDAMP are highlighted by applying arithmetic operations to all corresponding signal intensities from whole raw datasets on a datapoint-by-datapoint basis. To facilitate this, the datasets are processed into rectangular matrices and normalized in terms of both migration time and signal intensities. The results are visualized on density plots (also referred to as color maps or heat maps) providing a global view of the differences between samples. The main features of the package are briefly outlined below. A detailed description of the implementation and usage is part of the online documentation [10].

### Preprocessing

Raw datasets are binned along the m/z dimension to a specified resolution upon loading. Baselines may be subtracted by fitting the individual electropherograms to any user specified function (first order polynomial by default) by robust nonlinear regression as described by Ruckstuhl *et al*. [11]. However, the regression is performed in a global fashion in our implementation. Following baseline subtraction, noise may be removed from individual electropherograms by leveling to 0 all signal intensities falling within a threshold. By default, the threshold is calculated for every electropherogram as a specific multiple (5) of a standard deviation of signal intensities from a specified region of the electropherogram where no signals are expected (1 – 3 min). The datasets may be smoothed by applying predefined or user specified smoothing filters to all electropherograms in the dataset. Additionally, the datasets may be cropped along both the m/z and migration time dimensions.

### Normalization

**Figure 1:**
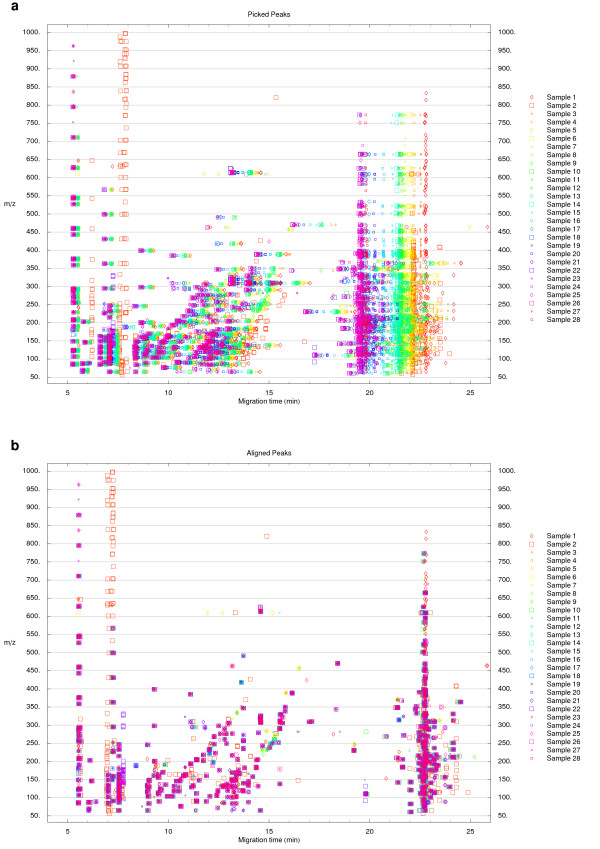
**Peak picking and migration time alignment**. (a) Visualization of the position of peaks picked from cation datasets acquired by CE-TOFMS. The data originates from a previous analysis of mouse liver extracts after treatment with acetaminophen [13]. (b) The peak sets were aligned to the peak set from Sample 1 using the alignment procedure described in the main text. The function derived by Reijenga *et al*. [14] for the normalization of migration times in CE was used as the time shift function.

Sample datasets are normalized to a reference dataset (e.g. one of the sample datasets) on a pairwise basis. For the purpose of the dataset alignment along the migration time dimension, a representative set of peaks is picked from all datasets using a modified Douglas-Peucker algorithm as described by Wallace *et al*. [12]. Raw migration times are used for all calculations. Using this approach, individual electropherograms are segmented into parts determined by strategic points. Initially, only the endpoints of an electropherogram are set to be the strategic points. Additional strategic points are assigned recursively to a datapoint in the electropherogram with the biggest orthogonal distance from a line connecting two neighboring strategic points. This distance must be above a specified threshold to avoid picking noise-related peaks. Vertical distance instead of an orthogonal distance is used in our implementation for the assignment of new strategic points. Strategic points with two neighboring strategic points having a smaller signal intensity are selected as peaks. The corresponding migration times may then be calculated as centroids from datapoints falling within a specified vicinity (time range) of the selected strategic points. The alignment of datasets along the migration time dimension is achieved by dynamic time warping (DTW) with an explicitly specified time shift (warping) function as described previously [13]. Any mathematical function can be specified as the time shift function. Optimal parameters for the time shift function are calculated for all sample datasets by combining dynamic programming (DP) and global optimization methods. The parameters of the time shift function are optimized to achieve the lowest sum of DP scores from all corresponding electropherograms. The DP scores are calculated by setting the partial scores to the distance between the two peaks of a subproblem (or a gap penalty value). The optimized time shift function is then used to rescale the timescale on the sample dataset (Figure 1). Signal intensities are adjusted to compensate for the compression or expansion of the peaks during time warping and thus conserve their areas. All electropherograms in the aligned sample datasets are interpolated and timepoints identical to those in the reference dataset are selected.

A standard library table (a list of m/z values and migration times for known compounds) may be aligned to the reference dataset using the same procedure. The aligned standard library table can later be used to annotate the plots or for the automatic localization of the peaks of the internal standard as described below.

Signal intensities in the sample datasets may be normalized according to a specified list of normalization coefficients (e.g. originating from the sample weights). Additionally, the signal intensities may be normalized according to the peaks of the internal standard. These peaks are then integrated in all datasets after alignment. The location of the peak of the internal standard in the reference dataset may be either specified explicitly or it may be extrapolated from the aligned standard library table. In the latter case, the user specifies only the name of the compound to serve as the internal standard.

### Result datasets

Following dataset normalization, various forms of direct comparisons may be performed to find differences between two or multiple datasets. Arithmetic operations or statistical tests are then applied to all corresponding signal intensities. The resulting dataset(s) has the same structure and dimensions as the compared datasets. Any processed, normalized, or result dataset can be easily exported as text or in binary format using Mathematica's built-in data export functionality.

To compare two datasets, the simplest way to highlight differences between them is to subtract the corresponding signal intensities (absolute difference). Alternately, dividing this difference by the larger of the two signal intensities provides a measure of the relative difference. Multiplying the corresponding signal intensities of the absolute and relative difference results highlights differences significant in both absolute and relative terms (absolute × relative difference). 

Outlier signals within a group of datasets may be located by calculating *z*-scores or by quartile analysis. A specified number of outliers may be removed from a set of corresponding signal intensities prior to *z*-score calculation. This limits the disproportionate influence of eventual outliers on the mean and standard deviation of the set which may lead to undesirably low *z*-score values. The result for the quartile-based analysis, an alternative to *z*-scores, is calculated as a difference between a specific signal intensity value and the third quartile (if it is smaller than the value) or the first quartile (if it is greater than the value) of the corresponding set, divided by the interquartile range. The result is set to 0 if the value lies between the first and the third quartile.

**Figure 2:**
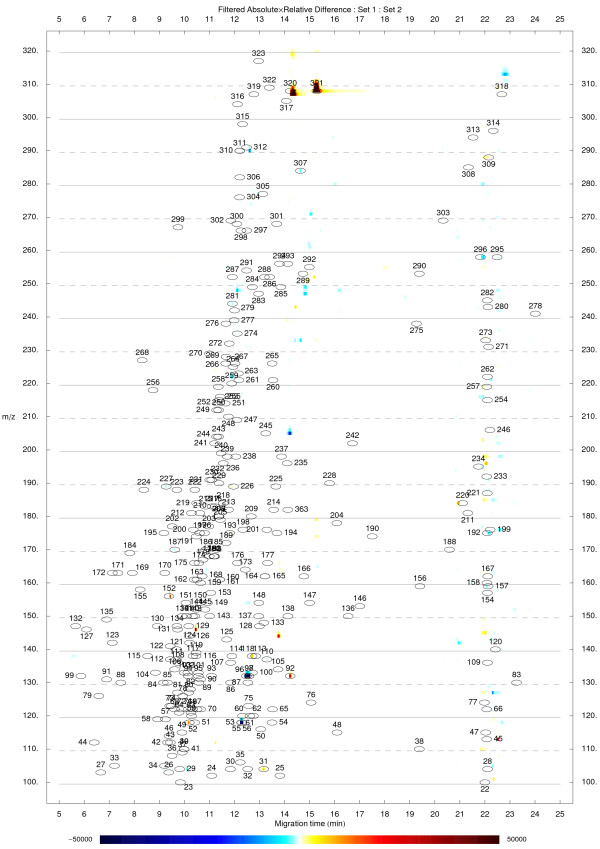
**Comparison of two groups of replicate datasets**. Visualization of an absolute × relative difference result between the averages of two groups of replicate cation datasets (n = 4). The result was further filtered as described in the main text with a *t*-score threshold of 3.71 (corresponding *p *= 0.01 when comparing two groups of four replicate values). The initial *t*-score dataset was smoothed by applying a moving average filter (window size 9) prior to filtering the absolute × relative result. Red color indicates signals with higher levels in Set 2, blue color indicates signals with lower levels in Set 2. The underlying datasets originate from previous work [13].

To compare two groups of replicate datasets, averaged datasets for both groups are calculated after normalization and these are used to generate the absolute, relative, and absolute × relative difference results. Additionally, *t*-scores are calculated for all groups of corresponding signal intensities. The resulting dataset may be used to directly identify the differences or to filter the absolute × relative result to remove statistically less significant differences (Figure 2).

An F ratio (one-way ANOVA) is used to find differences between multiple groups of replicated datasets. The F ratio, *t*-score, *z*-score, and quartile-based result datasets may be smoothed to suppress signals resulting from individual coinciding noise-related signal intensities. Details and template notebooks for the differential analysis approaches described above are part of the online documentation [10]. Additionally, any custom function to process corresponding signal intensities from datasets under comparison may be defined to highlight any difference or any pattern of interest.

### Visualization

**Figure 3:**
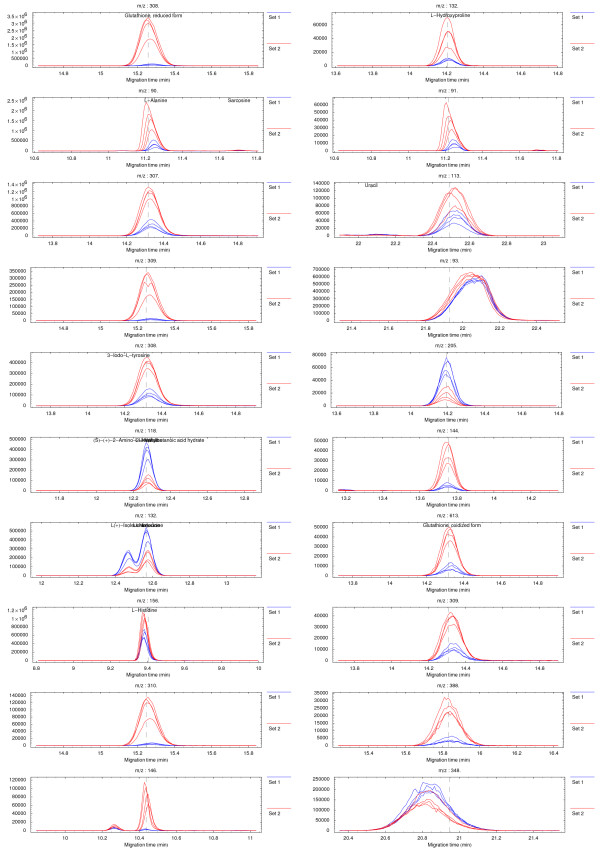
**Visualization of candidate differences as extracted ion electropherograms**. Overlaid electropherograms from aligned and normalized datasets corresponding to the vicinities of the 20 most significant differences in the dataset shown in Figure 2. The underlying datasets originate from previous work [13].

The sample datasets and the results of differential analyses are visualized using density plots. The axes represent the migration time and m/z values. Peaks appear as color-coded spots characteristic of the signal intensity (Figure 2). Multiple datasets may also be interlaced into each other so that electropherograms corresponding to the same m/z value appear next to each other on density plot (parallel plot) allowing to simultaneously explore the whole profiles. For any of the different types of results, plots displaying overlaid electropherograms for time ranges adjacent to the candidate differences can be generated in a descending order of significance to facilitate visual inspection and identification of false positives (Figure 3). The aligned standard library table may be used to place annotation labels directly on the density plots and electropherograms to allow easier identification of metabolites (Figure 2, 3).

## Results and discussion

### Preprocessing

The binning of raw datasets along the m/z dimension facilitates their processing into a rectangular matrix format. For datasets with high resolution along the m/z dimension (such as those originating from TOF instruments), binning provides a significant decrease in size and resolution suitable for visual inspection. Choosing a wider bin size may, however, lead to an undesirable dilution of weak signals in noise. This can be overcome by first performing the baseline subtraction and noise removal on datasets binned using a narrow binning window. The resulting datasets may then be binned to a resolution suitable for visual inspection.

### Normalization

A representative set of peaks is picked from the datasets for the purpose of migration time alignment. We modified the peak picking algorithm described above to use the maximum vertical distance from the line connecting two neighboring strategic points as a criteria for a new strategic point. This was done to avoid the necessity to normalize the migration time scale and the signal intensity scale prior to peak picking. However, by using the vertical distance, many datapoints within a peak fulfill the criteria of being above the vertical distance threshold. Excessive strategic point selection is suppressed by specifying a minimum distance between neighboring strategic points. The minimum distance is, by default, set to a quarter of a typical peak width so that the excluded time window in the vicinity of strategic points corresponding to the peak top and to the peak base partially or almost completely overlap.

Results of the migration time normalization/alignment are shown in Figure 1. As can be seen, the migration time shifts are significant between the original samples and the trend toward larger shifts with increasing migration time is apparent (Figure 1a). The quality of the overall alignment procedure can be seen in Figure 1b. The quality of the alignment is rather uniform over time. Isolated symbols do not correspond to misaligned peaks but rather to peaks higher than the peak picking threshold present only in certain datasets.

The alignment procedure described above proved robust to the presence of a large number of non-corresponding peaks between two aligned datasets. A small number of corresponding peaks picked from the datasets proved sufficient to find the optimal parameters of the time shift function. Given the robustness of the alignment procedure, missing peaks or erroneous peak picking do not significantly affect the quality of the alignment.

An iterative two-step alignment procedure proved beneficial for datasets with significant time shifts between corresponding peaks. To achieve a good alignment, a small gap penalty value is desirable to limit the number of non-corresponding peaks that are close enough in corresponding electropherograms to fall within the gap penalty and thus affect the alignment. However, when a small gap penalty is used for the alignment of datasets with large time shifts, the optimization procedure may not find the region of convergence to the global optimum. Therefore, a bigger gap penalty value is used first to generate an approximate alignment. The second alignment is then performed with a smaller gap penalty value starting with the parameters of the time shift function obtained from the primary approximate alignment. The DTW implementation of MathDAMP employs explicit time shift function specification. Generic time shift functions (such as polynomials or splines) may be specified for dataset alignment. Alternately, time shift functions incorporating a priori knowledge about the expected time shifts, as for example migration time normalization function for CE [14], may be used. Using explicit time shift functions provides the ability to control the flexibility or the rigidity of time warping. This may prove beneficial for some applications as improvement of alignment results of unconstrained DTW [15], by introducing rigid slope constraints, was reported [16]. Additionally, other existing alignment algorithms [5,17-20], that could also be implemented in MathDAMP, may hold advantages for specific applications.

As described above, the method to identify differences in profiles is not based on integrated peak lists and thus avoids common quantitative errors introduced by this task. It is important to realize that the described peak picking is used only for the purpose of alignment which as we described is very robust to possible peak picking errors.

### Data visualization

The density plot visualizations provide an overall intuitive view of the differences between samples (Figure 2). Peaks appear as colored spots of intensity corresponding to the magnitude of the differences between samples/groups. As described above, multiple alternative approaches for highlighting a difference of interest are available. Since different approaches may possess different strengths and weaknesses, evaluating them in parallel decreases the chance of missing an important difference.

The proper alignment of the datasets is a necessary prerequisite to obtain clear results. Misaligned peaks can lead to ambiguous signals on the density plots (e.g. appearing as doublets of opposite polarity red-blue) but these fortunately can be ruled out as false positives by visually exploring the overlaid electropherograms of the top candidate differences (Figure 3). The confirmation plots are thus an essential and easy way to identify false-positive signals since at this point there are no simple means to automate this process. Specific sources of ambiguous signals and possible ways to suppress their occurrence, as well as potential strengths and weaknesses of alternative approaches, for different kinds of differential analysis, are described in more detail in the respective example notebooks which are part of the online documentation [10].

The ability of MathDAMP to identify specific differences in complex metabolite profiles has been successfully demonstrated, leading to the discovery of a biomarker for liver oxidative stress [13] as well as facilitating enzyme activity detection and discovery in known and non-characterized proteins using non-targeted CE-MS analysis of complex substrate cocktails [21]. Overall, the MathDAMP tools can be seen as complementary to other methods that make use of integrated peak lists to find differences in profiles using downstream multivariate analysis. While both approaches may have limitations, MathDAMP can considerably simplify differential analysis of metabolite profiles that are very similar and is very robust to widely varying migration times and irregular peak shape.

## Conclusion

The MathDAMP package is capable of highlighting differences between complex metabolite profiles according to various criteria in an automated fashion. Since the whole (preprocessed and normalized) raw datasets are compared, the possibility of loss of information (e.g. due to common but unavoidable mistakes in peak picking or peak identification) is limited. MathDAMP differs from most other existing tools by combining very robust migration time normalization with a point-by-point approach to the identification of differences in profiles, facilitates the identification of metabolites, and provides multiple different ways in which data and differences in profiles can be visualized and analyzed. Moreover, the open architecture of the MathDAMP modules allows user to adjust multiple options to fit particular purposes or type of analytical method and offers extensive customizability for any user willing to manipulate the Mathematica code and potentially quickly implement any desirable new features. The current release does not provide quantification of compounds per se, something that is planned for future development. However, it is especially well-suited to highlight subtle differences between complex metabolite profiles.

## Availability and requirements

• **Project name: **MathDAMP

• **Project home page: **

• **Operating system(s): **Platform independent

• **Programming language: **Mathematica

• **Other requirements: **Mathematica 4.2 or higher

• **License: **free

## Abbreviations

CE – capillary electrophoresis

DP – dynamic programming

GC – gas chromatography

LC – liquid chromatography

MS – mass spectrometry

TOFMS – time-of-flight mass spectrometry

## Authors' contributions

MR conceived the approach of visualizing direct comparisons between raw metabolite profiles to highlight specific differences. RB, HK, TS, TN, MR, and MT suggested desired features and algorithmic approaches. RB carried out the implementation. HK, NS, MS, TS, and MR provided experimental data and supported the evaluation of the package. The online documentation and the manuscript were written by RB and MR with inputs from all co-authors.
